# Neurogenic Bladder and Acute Kidney Injury in Leukodystrophy

**DOI:** 10.7759/cureus.8707

**Published:** 2020-06-20

**Authors:** Zeki Kemec, Cevat Tüzün, Ali Gürel

**Affiliations:** 1 Nephrology, Batman District State Hospital, Batman, TUR; 2 Radiology, Batman District State Hospital, Batman, TUR; 3 Nephrology, Fırat University, Elazığ, TUR

**Keywords:** leukodystrophy, neurogenic bladder, acute kidney injury

## Abstract

Leukodystrophies are genetic white matter disorders. In the young, they represent an important cause of progressive neurological disability. Impairment of the bladder function may be part of the clinical picture of leukodystrophies. A neurogenic bladder is a dysfunctional urinary bladder caused by a disease of the central or peripheral nervous system involved in the control of micturition. In our patient, leukodystrophy-induced neurogenic bladder and acute kidney injury were revealed. If untreated, a neurogenic bladder can cause renal failure and urinary incontinence. Patients with a neurogenic bladder should be monitored, and management should aim to preserve renal function and achieve social continence.

## Introduction

The term ‘leukodystrophy’ refers to the deterioration of the white matter of the brain. The deterioration coincides with a clinical regression of skills and, in the most severe cases, neurological devastation. Leukodystrophies are genetic diseases with the degeneration of myelin sheaths in the central nervous system (CNS) and sometimes also in peripheral nerves. Although each of these diseases is individually quite rare, collectively leukodystrophies may affect as many as one in 7500 people [1-3].

Acute kidney injury (AKI) is defined as an abrupt or rapid decline in glomerular filtration rate. AKI may be caused by multiple etiologies, but it is associated with uremia when a rapid rise in urea or creatinine occurs. AKI may be classified into three general categories as follows: prerenal, intrinsic, and postrenal. Mechanical obstruction of the urinary collecting system, including the renal pelvis, ureters, bladder, or urethra, results in obstructive uropathy or postrenal AKI [4]. Neurogenic bladder is a dysfunctional urinary bladder caused by a disease of the central or peripheral nervous system involved in the control of micturition [5]. Impairment of bladder/bowel function may be part of the clinical picture of leukodystrophies [6].

Although the association of leukodystrophy with bladder dysfunction is mentioned, we have not found any case in the literature. Therefore, we believe that the case we present is important.

## Case presentation

The patient was a 35-year-old female, the third-born to healthy, non-consanguineous parents with a negative family history of neurologic disorders. The patient had no history of drug, alcohol, or cigarette use. She was admitted to our emergency department. Laboratory results were as follows: urea: 155.8 mg/dL, creatinine (Cre): 7.08 mg/dL, white blood cell (WBC): 14.8 x109/L, C-reactive protein (CRP): 197.7 mg/L, pH: 7.27 mm/h, partial pressure of carbon dioxide (pCO2): 32.2 mmHg, bicarbonate (HCO3): 14.5 mmol/L (Table 1). The patient was hospitalized in the internal medicine service with the diagnosis of acute renal failure and hydrated for three days. The Foley catheter was removed and discharged when the kidney tests become normal. She was admitted to our emergency department with the inability to urinate again. There were no accompanying neurologic signs in the overall clinical status. Her kidney tests were high (urea: 155.8 mg/dL, creatinine (Cre): 7.08 mg/dL, Table 1). The urinalysis was accompanied by urinary tract infection findings. Urine culture was obtained. She had acute renal failure. We put her in the nephrology service for examination and treatment. A Foley catheter was inserted. After the catheter was applied, about 2000 cc of urine was drained. We thought of bladder dysfunction. A 250 cc/h fluid replacement was performed. Ciprofloxacin 200 mg/day was started intravenously. Renal functions returned to normal three days after hydration. The urinary tract infection regressed. No growth was detected in the urine culture. On urinary ultrasonography, the renal parenchyma and ducts were normal. No mechanical obstruction was detected.

**Table 1:** Laboratory data of the patient ^x^First hospitalization ^y^Second hospitalization Cre: creatinine, Na: sodium, K: potassium, CL: chlorine, Ca: calcium, P: phosphorus, WBC: white blood cell, CRP: C-reactive protein, HCO3: bicarbonate Note: Since some data were not analyzed on some days, data were not entered in some cells of the table.

Date	One day^x^	Two day	Three day	Seven day^y^	Eight day	Ten day	Reference range
Glucose	100		92	106			70–10^5^ mg/dL
Urea	155.8	78.4	28	55.4	34.3	19	8–50 mg/dL
Cre	7.08	2.06	0.72	3.32	1.31	0.67	0.05–1.3 mg/dL
Na	133			128			132-150 mmol/L
K	4.1						3–5.5 mmol/L
Cl	102						90-115 mmol/L
Ca	9						8.3–10.6 mg/dL
P	4.4						2–6 mg/dL
WBC	14.8	8.6	5.5	11.3		5.1	3.5–11x10^9^/L
CRP	197.7	189.2	87.5	144.9	148.2	15.5	0-10 mg/L
pH	7.27	7.37	7.43	7.47			7.35-7.45
HCO3	14.5	17.8	20.2	21.8			22-30 mmol/L

**Figure 1:**
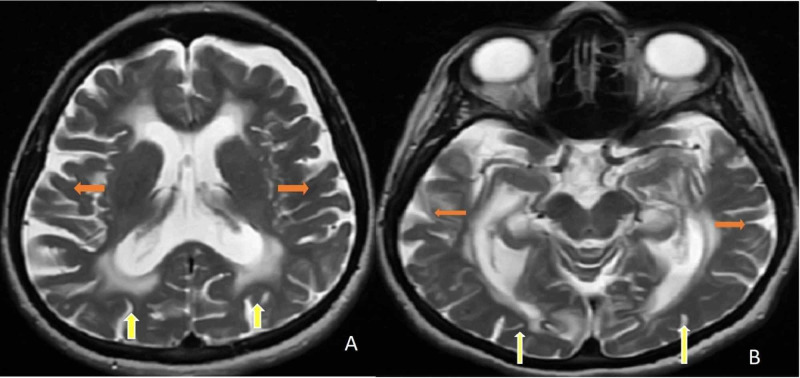
A-B. Axial T2 brain MRIs demonstrate bilateral periventricular, subcortical frontal, and parieto-occipital white matter involvement (yellow arrows). Axial T2 brain MRIs show atrophy of the cerebrum (orange arrows) with hyperintensities in the bilateral periventricular and subcortical white matter (yellow arrows). MRI: magnetic resonance imaging

**Figure 2:**
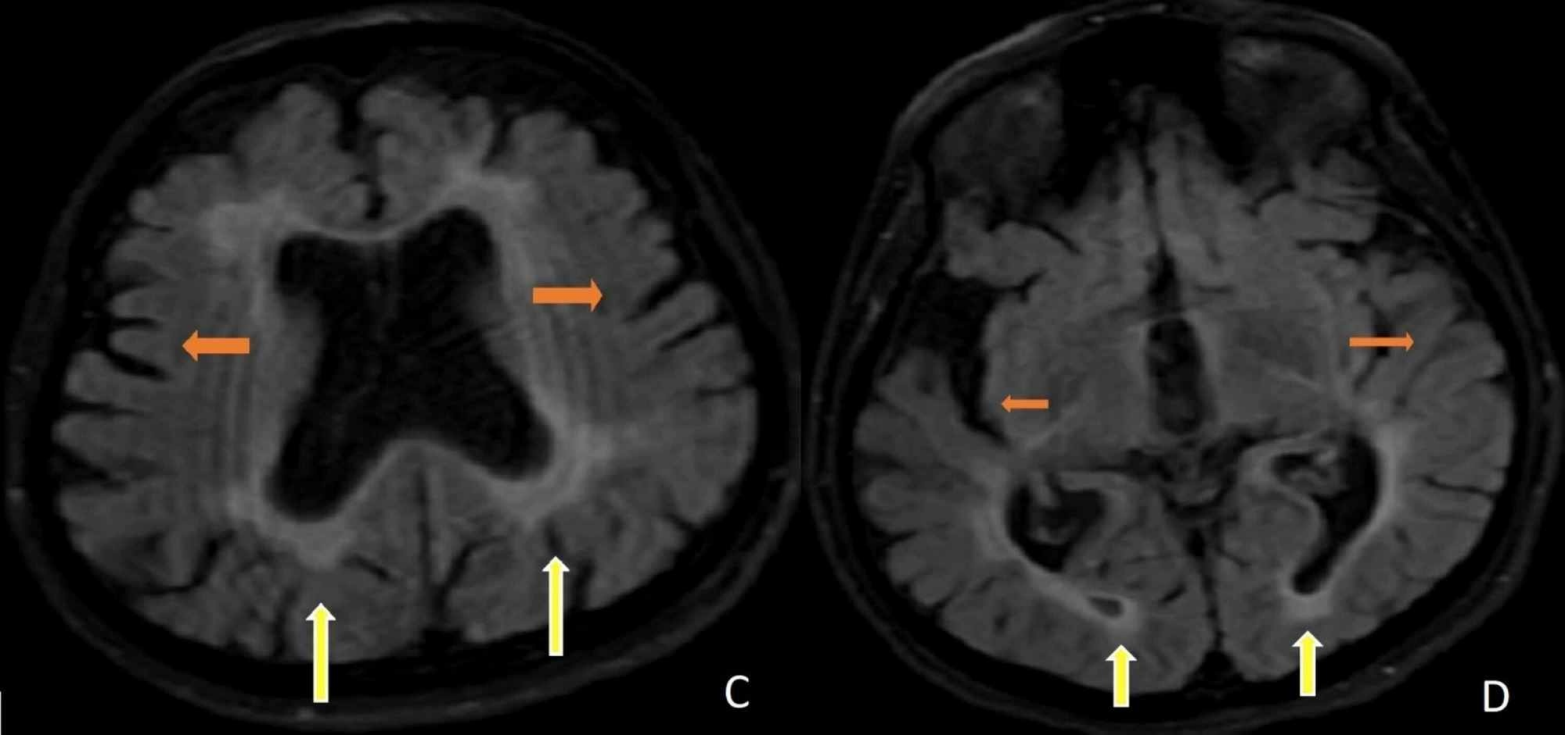
C-D. Axial FLAIR brain MRIs demonstrate diffuse hyperintensities in the periventricular and subcortical white matter (yellow arrows) with cerebral atrophy (orange arrows). FLAIR: fluid-attenuated inversion recovery; MRI: magnetic resonance imaging

During the follow-up, we observed that the patient had a bladder globe when the Foley catheter was removed. It was thought that there might be autonomic neuropathy or peripheral nerve damage. Urodynamic tests supported the neurogenic bladder. Urodynamic tests (uroflow study, postvoid residual volume, filling voiding cystometry, abdominal leak-point pressure, and external sphincter electromyography (EMG)) revealed decreased bladder capacity with preserved sensation, uninhibited detrusor muscle contractions, and detrusor-external sphincter synergy while sphincter EMG recorded normal amplitudes and duration of muscle potentials. The urologist suggested clean intermittent catheterization (CIC). Brain-cervical-thoracic magnetic resonance imaging (MRI) was performed. Diffuse involvement (leukodystrophy) was detected in the figures. In Figure 1 (A-B), axial T2 brain MRIs demonstrate bilateral periventricular, subcortical frontal, and parieto-occipital white matter involvement. Axial T2 brain MRIs show atrophy of the cerebrum with hyperintensities in the bilateral periventricular and subcortical white matter. In Figure 2 (C-D), axial flair brain MRIs demonstrate diffuse hyperintensities in the periventricular and subcortical white matter with cerebral atrophy.

## Discussion

Most leukodystrophies manifest themselves during childhood or adolescence, are incurable, and have a progressive course, leading to premature death. Diagnosis is important, as palliative or experimental therapies may offer benefits for reproductive counseling and family screening of currently unaffected individuals [1]. Autosomal dominant leukodystrophy with an autonomic disease is a slowly progressive disorder of the central nervous system (CNS) white matter characterized by the onset of autonomic dysfunction in the fourth to fifth decades, followed in months to years by pyramidal and cerebellar involvement. Autonomic dysfunction can include bladder dysfunction, constipation, postural hypotension, feeding difficulties, erectile dysfunction, and (less often) impaired sweating [7].

There was no history of spinal cord trauma in our patient. She did not have a metabolic disease like diabetes mellitus nor postrenal pathology. There was no other disease that could cause neurogenic bladder. In our patient, the neurogenic bladder and associated AKI was attributed to leukodystrophy. MRI and urodynamic studies supported this. She was discharged with CIC.

Making the diagnosis of a leukodystrophy requires knowledge of clinical features and neuro-imaging. Familiarity with the typical age of onset of various phenotypes of leukodystrophies, as well as heightened vigilance to brain MRI patterns, is invaluable in the diagnostic algorithm. In addition, electrophysiological findings, specific laboratory tests, and other special investigations may be needed to arrive at a precise diagnosis [1]. An MRI of the brain and spinal cord is the most important ancillary test in a patient suspected of having a leukodystrophy. The minimal requirement for a standard investigation is T1- and T2-weighted and ﬂuid attenuated inversion-recovery (FLAIR) images [1]. The field of leukodystrophies is growing owing to advances in MRI diagnostics and an explosion of gene discovery over the last decade. The study of leukodystrophies remains challenging owing to the limited number of study subjects for clinical trials. The nature of leukodystrophies lends itself to the use of MRI as a biomarker [1].

Bladder storage and voiding of urine are, respectively, under sympathetic (T10-L2) and parasympathetic (S2-S4) nervous system control. Therefore, the autonomic nervous system controls bladder function while the somatic nervous system controls the external urinary sphincter, allowing continence [5]. A neurogenic bladder is caused by a spinal reﬂex arc that occurs when the bladder becomes autonomous from higher centers [5]. CIC, the mainstay of treatment, is most suitable for resource-poor settings because it is eﬀective and inexpensive. Antimuscarinic drugs, such as oxybutynin, complement CIC by reducing detrusor overactivity. An intravesical injection of Botox and bladder augmentation surgery is required by a small subset of patients who fail to respond to combined CIC and oxybutynin therapy [5].

## Conclusions

There is theoretical information about the bladder involvement of leukodystrophy. But we couldn't find information that could correlate neurogenic bladder and urinary tract infection. Although we analyzed the literature extensively, we could not reach a similar case report. Thus, to our knowledge, this is the first and an unprecedented case in the literature. When anuria and acute renal failure occur in young patients, clinicians may consider leukodystrophic neurogenic bladder.

## References

[1] Kohlschutter A, Eichler F (2011). Childhood leukodystrophies: a clinical perspective. Expert Rev Neurother.

[2] Gordon HB, Letsou A, Bonkowsky JL (2014). The leukodystrophies. Semin Neurol.

[3] Waldman AT (2018). Leukodystrophies. Continuum (Minneap Minn).

[4] (2018). Acute kidney injury. http://emedicine.medscape.com/article/243492-overview.

[5] Maison POM, Lazarus J (2017). The management of paediatric neurogenic bladder: an approach in a resource-poor setting. Paediatr Int Child Health.

[6] Potic A, Popovic V, Ostojic J (2015). Neurogenic bladder and neuroendocrine abnormalities in Pol III-related leukodystrophy. BMC Neurol.

[7] Nahhas N, Rasekh PS, Vanderver A (2016). Autosomal dominant leukodystrophy with autonomic disease. GeneReviews® [Internet].

